# Preserving the Poly(A) Tail: Strategies Viruses Use to ‘CYA’ (Cover Your A’s)

**DOI:** 10.3390/v18010090

**Published:** 2026-01-09

**Authors:** Jeffrey Wilusz

**Affiliations:** 1 Department of Microbiology, Immunology and Pathology, Colorado State University, Fort Collins, CO 80523, USA; jeffrey.wilusz@colostate.edu; 2 Department of Environmental and Radiological Health Sciences, Colorado State University, Fort Collins, CO 80523, USA

**Keywords:** polyadenylation, deadenylation, terminal nucleotidyl transferases, viral mRNA stability

## Abstract

The poly(A) tail on viral mRNAs plays an important role in gene expression, given the role of the 3′ mRNA tail in mRNA stability and translation. Viruses have developed several strategies to maintain the integrity of their poly(A) tails. These include attracting stabilizing proteins through elements in the 3′ untranslated regions of their mRNA, remodeling their poly(A) tails using terminal nucleotidyl transferases, and blocking deadenylase access to the terminal 3′ end of their poly(A) tails using protein–protein interactions or through triple helical RNA structures. Collectively, the presence of these multiple strategies illustrates the vital overall need for viruses to maintain and preserve their poly(A) tails, highlighting a potential avenue for broad-spectrum antiviral development. In addition, poly(A) tail preservation strategies used by viruses may also be applied to RNA vaccines and therapeutics.

## 1. Introduction

**Figure 1:**
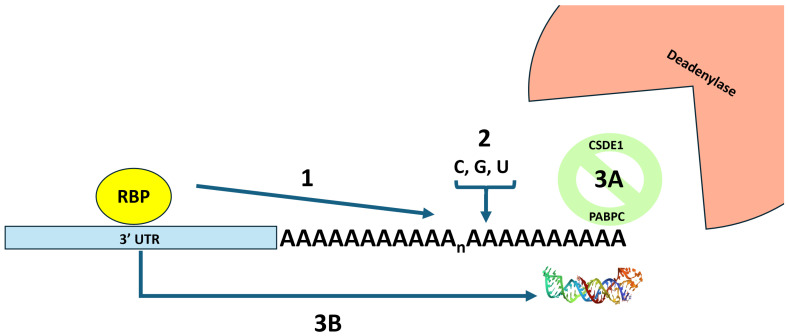
An overview of three strategies used by viral mRNAs to block deadenylation and preserve the integrity of their poly(A) tails. In strategy 1, an RNA binding protein (RBP) interacts with the 3′ UTR of the mRNA and prevents deadenylation by preventing the binding of proteins that promote deadenylation. In strategy 2, terminal nucleotidyl transferases are recruited that remodel the poly(A) tail by adding non-adenosine residues that confound the action of the largely adenosine-specific 3′ → 5′ deadenylases. In strategy 3, proteins or RNA structures are added to block the end of the poly(A) tail from deadenylases. Strategy 3A depicts the CDSE1 protein interacting with PABC1 and blocking deadenylase access. Strategy 3B depicts an RNA triple helix between the poly(A) tail and elements in the 3′ UTR of the mRNA that blocks deadenylase access. The picture of the RNA triple helix was derived from pdb_00006svs.

The presence and integrity of a poly(A) tail is vital for nearly all cellular mRNAs [1,2]. It is not surprising, therefore, that many viruses have chosen a strategy to incorporate a poly(A) on the 3′ end of their transcripts. The poly(A) tail plays a key role in nuclear export [3], translation efficiency [4], and transcript stability [5,6]. The process of deadenylation, or removal of the poly(A) tail, is the first canonical step in mRNA degradation [5,6]. Thus, maintenance of the poly(A) tail is a key consideration for viral mRNAs that use this strategy in their gene expression [7]. The poly(A) tail is coated with poly(A) binding proteins, most notably PABC1, which serve to protect the tail from random nucleases, interact with the translation initiation machinery at the 5′ end of the transcript, and communicate with the major CCR-NOT deadenylase when the mRNA is targeted for degradation [1]. The goal of this review is to highlight three key approaches that viruses utilize to stabilize and maintain the poly(A) tails of their mRNAs to promote efficient viral gene expression and replication. An overview of these approaches is presented in Figure 1.

## 2. The Canonical Approach: The Inclusion of 3′ UTR Elements to Attract RNA Binding Proteins to Prevent Deadenylation of Viral mRNAs

It has been well-documented that cellular mRNAs employ a strategy of recruiting RNA binding proteins (RBPs) via 3′ UTR elements to promote transcript stability [8]. It is not surprising, therefore, that this RBP recruitment strategy has also been reported for the stabilization of viral mRNAs. Since this strategy has been extensively reviewed (e.g., [7,9,10]), we will only provide an overview here by focusing on one protein—the cellular HuR/ELAV1 protein, the best characterized mRNA stabilizing factor to date [11].

Previous work from our laboratory demonstrated that numerous alphaviruses possess high-affinity binding sites for HUR/ELAV1 in their 3′ UTR that cause the cellular protein to re-localize from the nucleus to the cytoplasm during infection and usurp the HUR/ELAV1 protein to stabilize alphaviral mRNAs by blocking deadenylation [12,13,14,15]. Furthermore, the HUR/ELAV1 protein was required for efficient viral replication [12,13,14,15]. Thus, a family of viruses clearly has evolved a conserved strategy to usurp the function of the HUR/ELAV1 protein to stabilize their poly(A) tails. The precise mechanism for how HUR/ELAV1 represses deadenylation has not been determined but likely involves preventing the binding of other proteins to AU-rich elements that promote deadenylation.

HUR/ELAV1 has also been shown to play a role in infections with other viruses—although not necessarily through preventing deadenylation or involving the poly(A) tail. HUR knockout was shown to block the replication of a flavivirus (Japanese encephalitis virus (JEV)) [16]. Hepatitis B Virus (HBV) mRNAs usurp HUR/ELAV1 to stabilize their RNAs and promote nuclear–cytoplasmic export [17]. Furthermore, targeting HUR/ELAV1 with the drug CMLD-2 restricts HBV replication. Hepatitis C virus (HCV) replication requires HUR/ELAV1 [18], and the protein displaces the PTBP protein to facilitate the binding of other proteins to the 3′ UTR [19]. Next, Ebola virus mRNAs have AU-rich elements (AREs)—which are potential HUR/ELAV1 binding sites—in their 3′ UTR that influence RNA stability [20]. Finally, HUR/ELAV1 can also exert its influence on the 5′ UTR of some viral RNAs. HUR/ELAV1 promotes translation of coxsackie virus B3 genomic RNAs (by displacing another RBP—PCBP-2—in the 5′ cloverleaf that promotes replication) [21]. HUR was also shown to bind to the internal ribosome entry site (IRES) of Enterovirus 71 and promote replication [22]. Therefore many different viruses have opted to target and usurp the function of the HUR/ELAV1 protein to promote their replication—and have taken advantage of the versatility of this cellular RNA binding protein to use it in a variety of fashions.

The HUR/ELAV1 protein, of course, is not the only cellular stabilizing factor that has been usurped by viruses to protect their transcripts from RNA decay. As a recent example, a group of plus-sense RNA viruses, including avian leukosis virus subgroup J (ALV-J), reticuloendotheliosis virus (REV), chicken astrovirus (CAstV), and porcine epidemic diarrhea virus (PEDV), was shown to bind Musashi homolog 1 (MSI1) in the 3′UTR of viral transcripts to provide stability of the RNAs during infection [23]. Several recent reviews are available for more information on host RNA binding proteins that are usurped by viruses (e.g., [24,25]).

## 3. TENTs Aren’t Just for Camping: Readenylation and Mixed Poly(A) Tails on Viral RNAs

The addition of non-templated nucleotides to the 3′ ends of mRNA is not the sole domain of nuclear polyadenylation. There is a surprisingly large number of terminal nucleotidyl transferases in host cells [26]. One key function of these enzymes is illustrated by the TRAMP complex which adds short adenosine tails to the 3′ end of structural RNAs to initiate their degradation by providing a landing pad for 3′ → 5′ exonucleases [27]. Terminal 3′ uridylylation by TUT4/7 also serve as a mark to promote RNA degradation [28]. Interestingly, a forward genetic screen in C. elegans identified a TUT4/7 homolog—CDE1 as an antiviral factor that can directly uridylate the 3′ end of Orsay virus genomic RNA [29]. Furthermore, in mammalian cells, RACE-seq studies coupled with TUT4/7 knockdowns have demonstrated that a large percentage of influenza mRNAs contain terminal uridylates as they are being labeled by the TUT enzymes for degradation [29]. Therefore, it appears that one strategy that host cells may attempt to use to thwart viral infections is the addition of non-templated residues to the 3′ end of viral mRNAs to target them for degradation.

Viruses, however (and perhaps not surprisingly in their ongoing molecular arms race with the host cell), have figured out a way to usurp the addition of 3′ non-templated RNA residues to their advantage. Cells contain a terminal nucleotidyl transferase called TENT4 that exists in multiple complexes, including the TRAMP complex mentioned above, as well as in the cytoplasm in complex with other RNA binding proteins, most notably ZCCH14 [28]. These cytoplasmic TENT4 complexes are known to naturally extend poly(A) tails on approximately a fifth of cellular transcripts [26,30]. Furthermore, TENT enzymes often incorporate non-adenosine residues into these extended tails [31,32]. Importantly, these non-adenosine tail insertions can significantly extend the half-life of mRNAs [33], stalling both the CCR4 and CAF1 enzymatic components of the CCR4-NOT deadenylase complex [34]. Mathematical modeling of in vitro deadenylation assays suggested that the insertion of non-A residues could slow down deadenylation rates ~6–11 fold, with pyrimidine insertions having the largest impacts [34]. The CCR4 enzyme, for example, has been shown to have a three-nucleotide pocket for nucleotide specificity [35] and hence the insertion of a non-A residue into a poly(A) tail would clear result in a pause at or around the unconventional position. Thus, if a virus could evolve a way to attract the ZCCH14-TENT4 complex to its transcripts, the RNAs could become significantly more stable by the addition of non-A residues to an elongated tail at their 3′ ends. Viruses have indeed done just that: to date, four independent viruses have been demonstrated to use this strategy to promote their gene expression and infections.

Hepatitis B virus (HBV), a major cause of chronic liver disease, is a 3.2 kb circular DNA virus that generates a series of mRNAs from four promoters [36,37]. Interestingly, during infection, HBV mRNAs have ~50% longer poly(A) tails than cellular mRNAs and HBV does not globally target cellular mRNAs for decay as is seen in other viruses [38]. HBV mRNAs, as it turns out, are major substrates for TENT4-mediated tailing in infected cells, and these extended poly(A) tails require a regulatory element in the 3′ UTR known as the PRE (post-transcriptional regulatory element). Mutational analysis identified a key stem with a CNGG(N) family loop confirmation (a single bulged G flanked by A helical regions) [38]. This stem loop conformation is very similar to a fundamental component of another hepatitis B-like virus (woodchuck hepatitis virus) PRE that is used in biotechnology to increase gene expression in lentivirus expression systems [39]. This stem loop element is a high-affinity binding site for the ZCCCH14 protein of the TENT4 complex. Intriguingly, HBV replication is also substantially blocked by a dihydroquinolizinone class drug called DHQ-1 or RG7834 [40,41]. This inhibitor was shown to work through the PRE [41], and three-hybrid analyses demonstrated that this small-molecule drug binds specifically to the TENT 4 enzyme family [40]. Thus, multiple independent lines of evidence indicate that the ZCCH14-TENT4 complex plays a key role in upregulating HBV gene expression and maintaining a productive infection through poly(A) tail remodeling.

Human cytomegalovirus (HCMV), a large DNA virus of the betaherpesvirinae subfamily, is a major pathogen in congenital infections and diseases of the immunosuppressed/immunocompromised [42]. Interestingly, HCMV remodels protein synthesis (the ‘translatome’) in infected cells without enhancing the decay of cellular mRNAs like other herpesviruses [43]. This over-stimulation of viral mRNA-directed protein synthesis relative to host cell proteins is strongly associated with an increased length of the poly(A) tail on HCMV transcripts relative to cellular mRNAs. One study demonstrated that HCMV mRNA poly(A) tails, for example, are ~50% longer than the tails found on host cell transcripts (127 vs. 86 bases) [44]. Three factors have been associated with these longer poly(A) tails during HCMV infection. First, CNOT 1 and CNOT3, structural components of the CCR4-NOT deadenylase complex [45], are dramatically upregulated during HCMV infection and are both strongly proviral [44]. This may indicate that the CCR4-NOT complex is altered in some way during infection to protect HCMV transcripts. Knockdown of CNOT1/3 has a major impact (a ~2000× decrease) on HCMV replication. CNOT1/3 do not have a similar impact on Herpes Simplex Virus 1 (HSV1) or vaccina virus infections, indicating specificity for HCMV mRNAs. Thus, alterations in the deadenylase complex appear to be one part of the strategy used by HCMV to preserve the poly(A) tail on its transcripts. Next, HCMV also induces the expression of Cytoplasmic Polyadenylation Element Binding protein (CPEB1) [46]), a protein known to recruit cytoplasmic poly(A) polymerase and increase poly(A) tail lengths of targeted mRNAs [47]. Depletion of CPEB1 causes a reduction in HCMV viral titers as well as less cytopathology associated with infections [46]. Thus, some HCMV mRNAs may usurp the CPEB1-associated cytoplasmic polyadenylation machinery (which contains the cytoplasmic poly(A) polymerase GLD2) to increase the poly(A) tails on some its mRNAs. Finally, the IE domain—a stem containing a penta-loop element similar to those found in the HBV PRE discussed above—has been characterized in HCMV RNA 2.7 that binds ZCCH14 in RNA pulldown studies and attracts TENT4 to elongate the poly(A) tail, occasionally with non-adenosine residues [38]. Curiously, the IE stem loop is located at the 5′ end of the HCMV transcript rather than the 3′ end near the poly(A) tail as the stem loop element is in HBV. This indicates that elements that attract the ZCCHC14-TENT4 complex to re-configure the poly(A) tail on an mRNA may do so in a position-independent fashion. In summary, the existence of this three-pronged strategy by HCMV to optimize and maintain the poly(A) tails of its mRNAs clearly suggests the importance of these non-templated 3′ residues on viral transcripts to the viral infection.

Aichi virus is an emerging human pathogen of the Kobuvirus genus of the *Picornaviridae* [48]. It is transmitted by the fecal–oral route and can cause gastroenteritis. A massively parallel approach to discover viral RNA regulatory elements identified a particularly robust ~120-base element called K5 in the 3′ UTR of Aichi virus that stimulates TENT4-mediated mixed tailing of transcripts [49]. Interestingly, complementary approaches identified a novel protein—ZCCHC2—that binds to the K5 element and recruits TENT4. The K5 element is necessary and sufficient to recruit TENT4 and stimulate gene expression as it can enhance gene expression when inserted into Adeno-Associated Virus (AAV) vectors. Therefore, not only is the strategy to attract TENT4-mediated mixed tailing being extended to multiple viruses in the RNA (and DNA) virus world but there now appear to be additional ways beyond ZCCHC14 to attract the enzyme to act on viral poly(A) tails.

Hepatitis A virus (HAV), a positive-sense RNA virus associated with hepatitis acquired by the fecal–oral route [50], is our latest addition to the growing list of viruses that utilize the TENT4-mediated mixed tailing strategy. Following CRISPR screening that demonstrated TENT4 to be a proviral host factor for HAV [51], ZCCHC14 and TENT4 have been shown to bind to the 5′ UTR and be required for efficient HAV infection as demonstrated by depletion and DHQ1/RG7834 drug treatment [52,53,54]. Curiously, while TENT4 clearly promotes HAV infection, it has been recently shown not to be associated with an increase in poly(A) tail length on full-length HAV mRNAs [55]. Rather, TENT4 was shown to add to the 3′ end of an HAV subgenomic RNA derived from the 5′ UTR that represents 1–3% of HAV transcripts during infection. While the source and role of this subgenomic 5′UTR-derived transcript is not clear, it may be a prematurely terminated transcription product or a stabilized degradation intermediate that clearly appears to have significant impact on HAV biology. Thus, HAV appears to have extended the strategy of using TENT4 to stabilize viral transcripts by usurping the cellular enzyme to stabilize a novel non-coding RNA.

The implications of these novel strategies to extend poly(A) tails is not limited to the field of virology. TENT-based adenylation of RNAs also has recently been shown to impact the exploding field of mRNA-based therapeutics. In macrophages, COVID mRNA vaccines, most notably the Moderna mRNA-1273 vaccine, are associated with the TENT5A poly(A) polymerase that is resident in the endoplasmic reticulum and undergo significant re-adenylation [56]. Thus, the deadenylation of cytoplasmic RNAs is clearly not irreversible. A thorough understanding of TENT-based mixed tailing will enhance the efficacy of mRNA vaccines and therapeutics, another example of how initial work in virology has broadened our understanding of practical and applied molecular biology.

## 4. Putting a Lid on Deadenylation: Possible ‘Capping’ of the Poly(A) Tail

The process of deadenylation requires that the poly(A) tail be accessible to the CCR4-NOT complex. Another way of preserving the tail on viral mRNAs would be to sequester the 3′ end of the viral mRNA poly(A) tail in some fashion to prevent access to the deadenylase. Two strategies along these lines have been demonstrated to date in viral infections.

The family of La-related proteins (LARPs), while perhaps best known for binding by the LARP 7 protein to the 3′ UUU motif on nascent RNA polymerase III transcripts and protecting the end from exonucleases [57], also play a role at the 3′ end of mRNAs. LARP1 and LARP 4 bind to both the poly(A) tail as well as PABPC1 (the cytoplasmic poly(A) binding protein) and protect mRNAs from degradation [58]. LARP4 has been shown to migrate from the nucleus to the cytoplasm during infection and facilitate the translation of mRNAs from the porcine epidemic diarrhea coronavirus (PEDV) in Vero cells as well as in an in vitro translation system [59]. LARP4 is also enriched in vaccinia virus (VACV) viral factories and facilitates efficient expression of VACV mRNAs [60]. The LARP story, however, particularly with LARP1, is complex. The LARP1 protein has also been shown, for example, to bind to 5′ m7Gppp caps and terminal oligopyrimidine motifs located in the 5′ UTR of mRNAs and regulate translation [61]. LARP1 does appear to have numerous interesting functions in viral infections beyond instances where it has been shown to function at the 3′ end of transcripts. Some enteroviruses, such as EV-D68, EV-A71, and coxsackie virus A16, cleave LARP1 to counteract its antiviral influence through binding to the 5′UTR of viral transcripts [62]. In hepatitis C virus (HCV), Vesicular Stomatitis virus (VSV), Middle East Respiratory Syndrome (MERS) coronavirus, and Chikungunya virus (CHIKV) infections, LARP1 appears to play a role in the endocytosis of viral particles as well as HCV viral spreading through RNA-dependent interactions with HCV core protein [63]. Finally, LARP1 strongly interacts with SARS-CoV-2 viral RNA and restricts replication [64]. Therefore, while LARP proteins clearly possess the ability to interact with PABPC and protect the poly(A) tail, their interplay with viruses is complex (similar to the multiple roles played by HUR/ELAV1 discussed above).

Cold Shock Domain Binding Protein E (CSDE1), also known an UNR (Upstream of N-Ras), was identified decades ago as required for efficient IRES-dependent translation in a variety of members of the Picornaviridae family, including poliovirus and human rhinovirus [65,66]. CDSE1/UNR has also been shown to be proviral for several other viruses including Human Immunodeficiency virus (HIV-1) [67], SARS-CoV-2 [64,68,69,70], snakehead vesiculovirus (SHVV) [71], and VSV oncolytic viruses [72,73]. Additional CSDE1/UNR interactions have been identified in the 3′ UTR of tick-borne encephalitis virus RNA [74] and Classical Swine Fever virus (CSFV) non-structural proteins [75,76]. In addition to its role in IRES-mediated translation, CSDE1/UNR was also demonstrated to interact with poly(A) binding protein (PABPC1) and play a role in regulating mRNA turnover [77,78]. Since PABPC1 can stimulate deadenylation by CCR-NOT, it is possible that proteins that interact with PABPC-1, such as CDSE1, eIF4G [79], and LARP4, could prevent that stimulation of poly(A) tail removal by PABPC-1 [80] and essentially block the 3′ end of the poly(A) tail from ready access by the 3′-5′ deadenylase.

In addition to using protein–RNA interactions to protect viral RNA poly(A) tails, the strategy of using RNA–RNA interactions has also been reported for a variety of non-coding transcripts [81]. The polyadenylated nuclear PAN RNA from Kaposi Sarcoma Herpesvirus (KSHV) has been shown to contain a 40 nucleotide U-rich loop that binds the poly(A) tail through the formation of a triple helix [82,83,84]. Triple helices have also been shown to stabilize the 3′ end of RNAs that lack a poly(A) tail [85,86]. In addition to classic viral RNAs, the capacity to form triple helices between 3′UTR elements and the poly(A) tail can be readily observed in many plant and fungal transposon RNAs [87]. For example, a retrotransposon called Evade also uses triple helix formation between the poly(A) tail and a 3′ UTR element to stabilize the transcript and prevent deadenylation [88]. An in-depth analysis of an additional element motif used by the rice hAT transposon mRNA to form a triple helix with its poly(A) tail has also been reported [89]. Finally, the formation of a pseudoknot structure between a 19nt conserved sequence element (CSE) and the poly(A) tail that is stabilized by base triples is required for the replication of Sindbis and Chikungunya viral RNAs [90]. A similar pseudoknot poly(A) tail - 3′ UTR element stabilized by base triples also appears to be a conserved feature of the pepino mosaic virus, a tomato plant pathogen, and its relatives [91]. Thus, the strategy of using triple helices to create a novel structure at the 3′ end that serves as an impediment to deadenylases and 3′ → 5′ nucleases is clearly at play in the virosphere.

In summary, all 3′-5′ exonucleases, including deadenylases, need to get a toehold onto a single stranded 3′ end of RNA to initiate decay. Therefore, in addition to the use of modified bases, the use of proteins or RNA structure by viral RNAs to block deadenylases from gaining a grasp on the poly(A) tail is also an effective viral tail preservation strategy that can be mimicked in RNA therapeutics [92,93].

## 5. Perspectives and Future Directions

There is a plethora of non-canonical poly(A) polymerases and terminal nucleotidyl transferases in cells [31,32,94]. Given the clear evidence discussed above that viruses have focused on usurping this group of enzymes to modulate their poly(A) tails, a broader understanding of the impact of TENTs in viral RNA biology will likely provide additional insights into virus–host interactions. The recruitment of these enzymes to act on viral transcripts, for example, may have negative impacts on cell biology as the TENTs are less able to perform their normal cell function if they are focusing on viral transcripts. There are, for instance, numerous examples of effects of the knockdown or overexpression of TENT proteins in cell and organism biology. TENT4A has properties of the tumor-suppressor [95], TENT5A knockout mice have severe skeletal deformities [96], and TENT5C knockdown increases B cell proliferation [97]. Therefore, it may be interesting to determine the contribution of the commandeering of TENT proteins by viral mRNAs on cytopathology and pathogenesis.

While having a poly(A) tail provides numerous benefits for gene expression and represents the cellular standard, the fact that several virus families (e.g., flaviviruses) have chosen to totally forego the use of a poly(A) tail on their mRNAs emphasizes the double-edged sword of possessing one. Poly(A)-independent gene expression clearly is a viable strategy for these viruses, as it is for cellular histone mRNAs [98,99]. For viral mRNAs that contain poly(A) tails, more work is clearly needed to fully define the regulation of the rate of deadenylation of viral mRNAs, fully appreciate the role of PABPC in viral RNA gene expression, understand cell-type-specific impacts on viral poly(A) tail metabolism, and better understand the mechanistic interplay between the viral poly(A) tail, PABPC, and cellular/viral RNA binding proteins (RBPs) on the body of the transcript. Our understanding of cellular messenger RNPs is currently rather rudimentary. Thus, additional work on understanding viral messenger RNPs will undoubtedly provide key insights into cellular molecular biology.

Viruses have likely learned many tricks to optimize the stability, expression, and impact of their ‘non-self’ transcripts in the cytoplasm. Therefore, investigations into viral mRNA–host cell interactions are likely to provide insight into the optimization of the design, delivery, and maintenance of mRNA vaccines and therapeutics [33]. As noted above, the highly successful Moderna mRNA-1273 vaccine transcript is associated with TENT5A that is resident in the endoplasmic reticulum and undergoes significant re-adenylation that enhances its efficacy [56]. Thus, additional insight into mechanisms for viral mRNA poly(A) tail preservation can serve as a foundation for the optimization of therapeutic mRNA designs.

## Data Availability

The original contributions presented in this study are included in the article. Further inquiries can be directed to the corresponding author.
